# Sea-level rise and storm surges structure coastal forests into persistence and regeneration niches

**DOI:** 10.1371/journal.pone.0215977

**Published:** 2019-05-02

**Authors:** William S. Kearney, Arnold Fernandes, Sergio Fagherazzi

**Affiliations:** Department of Earth and Environment, Boston University, Boston, Massachusetts, United States of America; Centro de Investigacion Cientifica y de Educacion Superior de Ensenada Division de Fisica Aplicada, MEXICO

## Abstract

The retreat of coastal forests as sea level rises is well documented; however, the mechanisms which control this retreat vary with the physical and biological setting of the interface between tidal marsh and forest. Tidal flooding and saltwater intrusion as well as flooding and wind associated with storms can kill trees. Even if these processes do not kill stands, they may halt regeneration because seedlings are more sensitive to stress. We present a case study of a coastal pine forest on the Delmarva Peninsula, United States. This forest contains a persistent but nonregenerating zone of mature trees, the size of which is related to the sea level rise experienced since forest establishment. The transgression of coastal forest and shrub or marsh ecosystems is an ecological ratchet: sea-level rise pushes the regeneration boundary further into the forest while extreme events move the persistence boundary up to the regeneration boundary.

## Introduction

As sea-level rise accelerates [1–3] and regimes of precipitation [4–7] and storms [8–11] change, the spatial distribution of coastal ecosystems will also change. Exactly how the patterns of salt marshes, mangroves, freshwater marshes, tidal forests and upland environments evolve depends on complex interactions between physical forcings (sea level, precipitation and storms) and ecological processes (e.g. growth, competition, regeneration) in each of these ecosystems. The retreat of coastal forests as sea level rises is well documented [12–16]; however, the mechanisms which control this retreat vary with the geomorphological, hydrological, and ecological setting of the marsh-forest interface. Trees can be killed by increased tidal flooding, either because they are not flood-tolerant or because they are not tolerant to the salinity of flood waters. Saltwater intrusion in which the groundwater table becomes more saline, whether due to sea-level rise or groundwater withdrawals, can also lead to forest decline [17]. Droughts can amplify these effects by reducing the supply of fresh water [14]. Even if these processes do not kill trees, they may halt the regeneration of the forest because seedlings can be more sensitive to environmental change and variability [13, 18–20]. At the same time, there exist physical mechanisms, such as fresh groundwater inputs from the upland, and ecological mechanisms, such as competition for light, which can slow or even stop forest retreat [13, 21–25]. There is therefore a distinction between the regeneration niche [26], in which seedlings are able to establish and the persistence niche [27], in which mature plants that have already established maintain their position. These niches manifest on a heterogeneous landscape as demographic patterns in vegetation zonation.

Here we describe a persistent but nonregenerating zone of coastal pine forest on the Delmarva peninsula, United States, the size of which is related to the sea level rise experienced since forest establishment. The characteristics of this zone suggest that the transition between coastal forest and shrub or marsh ecosystems is an ecological ratchet [28]: gradual sea-level rise pushes the lower boundary of regeneration further into the forest while extreme events move the lower boundary of the persistent zone up to the regeneration boundary. Up to 20% of the forested land area in the Eastern Shore of Virginia could lie within this persistent zone and is therefore vulnerable to permanent forest dieback following an extreme storm.

## Materials and methods

### Site description

**Fig 1:**
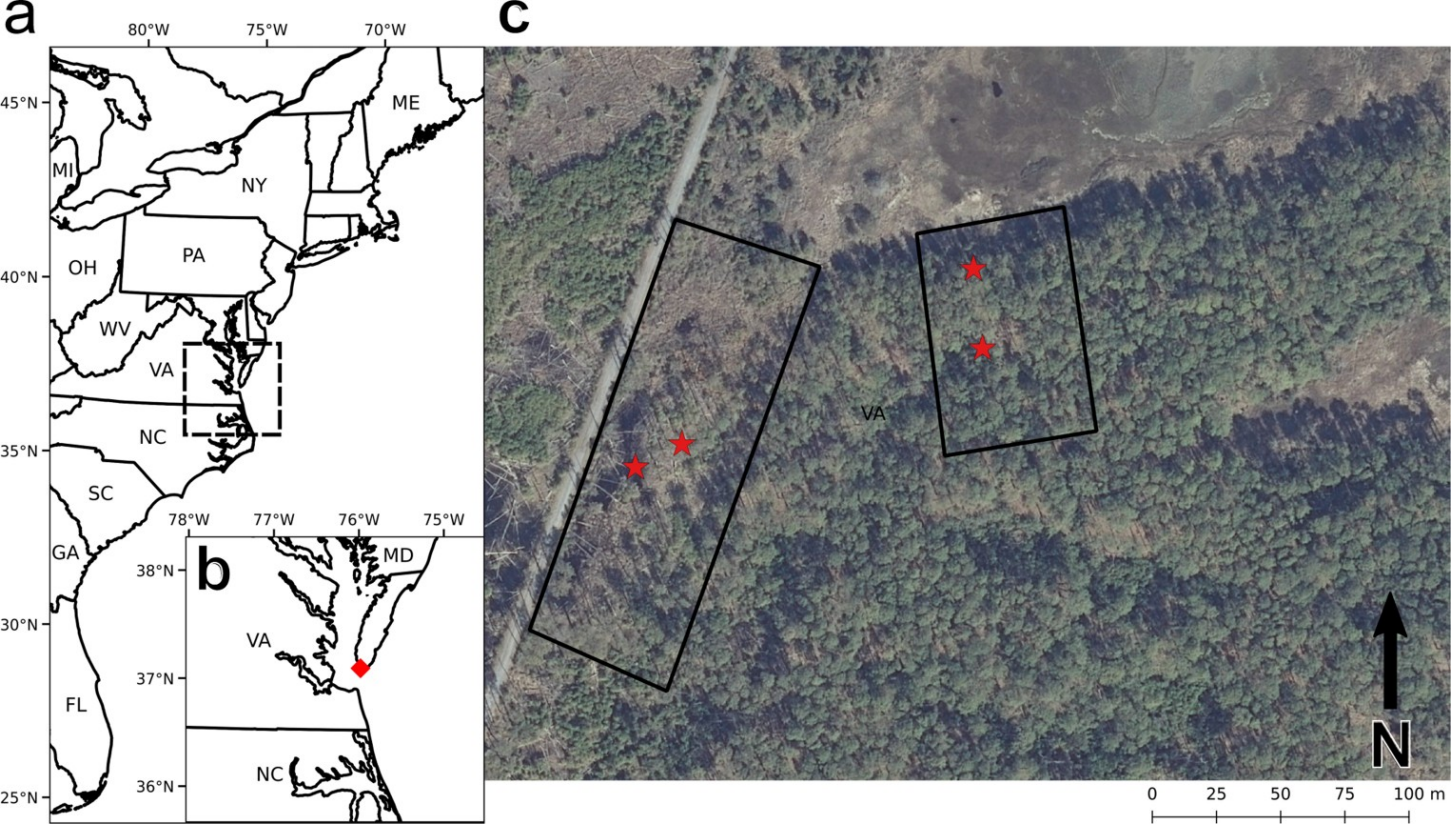
Site map. (**A**) The location of the site (red star) within the mid-Atlantic region of the United States. (**B**) The study site. The hollow site (left) and hillslope site (right) are outlined. The groundwater well locations are given by red stars. High-resolution orthoimagery courtesy of the United States Geological Survey (image date: February 14, 2013).

We examine the relationship between topography, hydrology and tree ecology in a loblolly pine (Pinus taeda) forest in the Eastern Shore of Virginia National Wildlife Refuge on the southern tip of the Delmarva Peninsula, United States (Fig 1). We worked at two sites within the Refuge, the "hillslope" site, which ascends from a salt marsh on its northern boundary into the pine forest, and the "hollow" site, next to Wise Point Road and characterized by a hummock-and-hollow microtopography with dry high points (hummocks) colonized by trees and flooded low points (hollows) covered with grasses. Permission to conduct this study within the Refuge was granted by the United States Fish and Wildlife Service.

On the hillslope, moving into the forest from the Spartina alterniflora salt marsh, one encounters a zone of woody shrubs (Juniperus virginiana, Iva frutescens, Baccharis halimifolia and Myrica cerifera) before entering the pine forest. The region of the forest closest to the salt marsh has an open understory with Smilax spp. and Andropogon spp. Moving further into the forest, a sharp boundary of pine saplings stands. Below this point, no young pine trees are found. Above this point, the understory is dominated by these young trees and shrubs.

The hollow site is filled with the snags of dead pine trees which exist both on the hummocks and in the hollows. The hollows are covered in various grasses, including Phragmites australis and Andropogon spp. and shrubs like those found in the shrub zone at the hillslope site. Living pine trees, both mature trees and saplings, occupy the hummocks only. No living pine trees are found in the hollows. Much of the observed mortality at this site appears to have been caused by Hurricane Isabel in September 2003 (S2 Fig).

### Groundwater wells

To develop a sense of the major hydrological processes occurring at each site, we installed two groundwater wells. In January 2014, one well (the "lower" well) was installed at the hillslope site just upslope of the lower boundary of the pine forest (Fig 1). The other well (the "upper" well) was installed at the line of saplings. A borehole was augured to a depth of approximately a meter, which was well below the water table when the wells were installed. The borehole was fitted with slotted well screen and instrumented with a conductivity-temperature-depth sensor (Schlumberger Water Services CTD-Diver). A barometric pressure sensor was deployed aboveground to correct the pressure transducer measurements for atmospheric pressure. Each well was surveyed and referenced to the North American Vertical Datum 1988 (S3 Table).

In January 2015, the wells and sensors were removed from the hillslope site and installed at the hollow site. The upper well was placed on one of the hummocks, surrounded by both pine saplings and mature surviving pine trees (Fig 1). The lower well was placed in one of the flooded hollows (Fig 1).

### Topographic surveys

We used a combination of a Real-Time Kinematic GPS (Topcon Hiper V) and a total station to survey the topographic position of living mature pine trees, saplings and dead trees at both the hillslope and hollow site. At the hillslope, the positions of 24 live trees along a transect from the marsh into the forest were recorded with the total station. Fourteen points along the lower edge of the sapling boundary were also recorded with the RTK GPS. The nominal accuracy of the GPS is 0.5 cm in the horizontal and 1.0 cm in the vertical. While the horizontal locations of these points are accurate, we found that the elevations for these GPS points are noisy because of multipath effects in the forest. As we do not have comparable total station data for the sapling boundary, we obtain the elevations of the live trees and the sapling boundary points by sampling a LiDAR-based digital elevation model [29](VITA, 2011) at their horizontal locations. The nominal accuracy of the LiDAR DEM is better than 19.8 cm. There is an approximately 15 cm offset between the DEM elevations and the GPS elevations; however, we will only use the DEM elevations to calculate differences between the elevations of live trees and saplings, so that this offset cancels out in the analysis. All reported elevations will be GPS elevations. At the hollow site, individual GPS points were recorded for all of the mature live trees and dead trees. The saplings at the hollow site cluster together on the hummocks, so the boundary of each of these clusters was sampled with the GPS rather than the individual trees.

### Sea level rise and persistent zone analysis

Cores were extracted from the 24 live trees at the hillslope site for dendroecological analysis. The ages of the trees were determined by counting the rings from the pith. For cores that did not reach the pith, the pith year was estimated following the method of Duncan [30], assuming concentric ring growth and determining the number of missing rings from the missing radius divided by the mean ring width Further methods and results of the dendroecological analysis can be found in [31]. We use the ages of the trees obtained from this analysis to determine the amount of sea level rise in the period since the forest was established. To determine when the current lower boundary of the forest was established, we choose eight trees closest to the salt marsh that make up that boundary. We sampled the sea level record from Sewell's Point, Virginia, at each of the years of establishment for those eight trees to obtain a distribution of possible sea levels when the lower forest boundary was established. We do not have ages for the saplings, but we assume that they were established after Hurricane Isabel, the most recent major hurricane which could have disturbed the forest and killed other pine saplings. We take a six year window (2000–2006) around Hurricane Isabel, which occurred in September 2003, and sample the sea level record at those years to obtain a distribution of possible sea levels when the saplings established.

We then estimate the elevation difference between the originally established forest and the new saplings at each of the two sites. We determine the elevations of a subset of the topographic data points collected in the survey. At the hillslope site, we select the elevations (determined by sampling the LiDAR as above) of the eight trees that make up the lower boundary of the forest as well as the points collected along the sapling boundary. Multiple mature trees or sapling boundary points may lie within one 3 x 3 m DEM cell, so we select only the unique elevation values for each of these, leaving us with 6 observations of elevation at the lower boundary of the forest and 9 observations at the sapling boundary. At the hollow site, we select the elevations of the dead trees in the hollows which are below an elevation threshold (0.98 m NAVD88) determined from a logistic regression (S1 Text) (N = 55) and the points collected from the boundary of the sapling clusters (N = 141).

We estimate the elevation differences for the three resulting data sets: sea-level between the time of establishment and Hurricane Isabel, the elevation of the eight boundary trees on the hillslope and the sapling boundary, and the elevation of the lowest dead trees in the hollows and the sapling boundaries. We use a Hodges-Lehmann estimator [32] as a robust, nonparametric estimate of the elevation difference for each data set. Each of the data sets (sea level, hillslope trees and saplings and hollow trees and saplings) consists of two sets of elevations of size m and n. All of the m x n possible differences between the two elevation sets are computed and the median of these differences is taken as the estimate of the elevation difference. From this procedure, we obtain a point estimate for the elevation difference as well as a 95% confidence interval about that estimate and compare the sea-level rise to the elevation difference between the lowest mature trees and the saplings at each site.

We use the LiDAR-based digital elevation model and the NOAA Coastal Change Analysis Program Land Cover classification from 2010 [33](NOAA 2012) to construct an estimate of the potential area of forest within persistence zones. We computed the average elevation on the digital elevation model for each land cover classification pixel and then calculated the number of forested pixels below the elevation range spanned by the Wise Point sapling boundaries at both the hillslope and the hollow site as determined by the RTK GPS surveys.

## Results

### Topographic surveys

**Fig 2:**
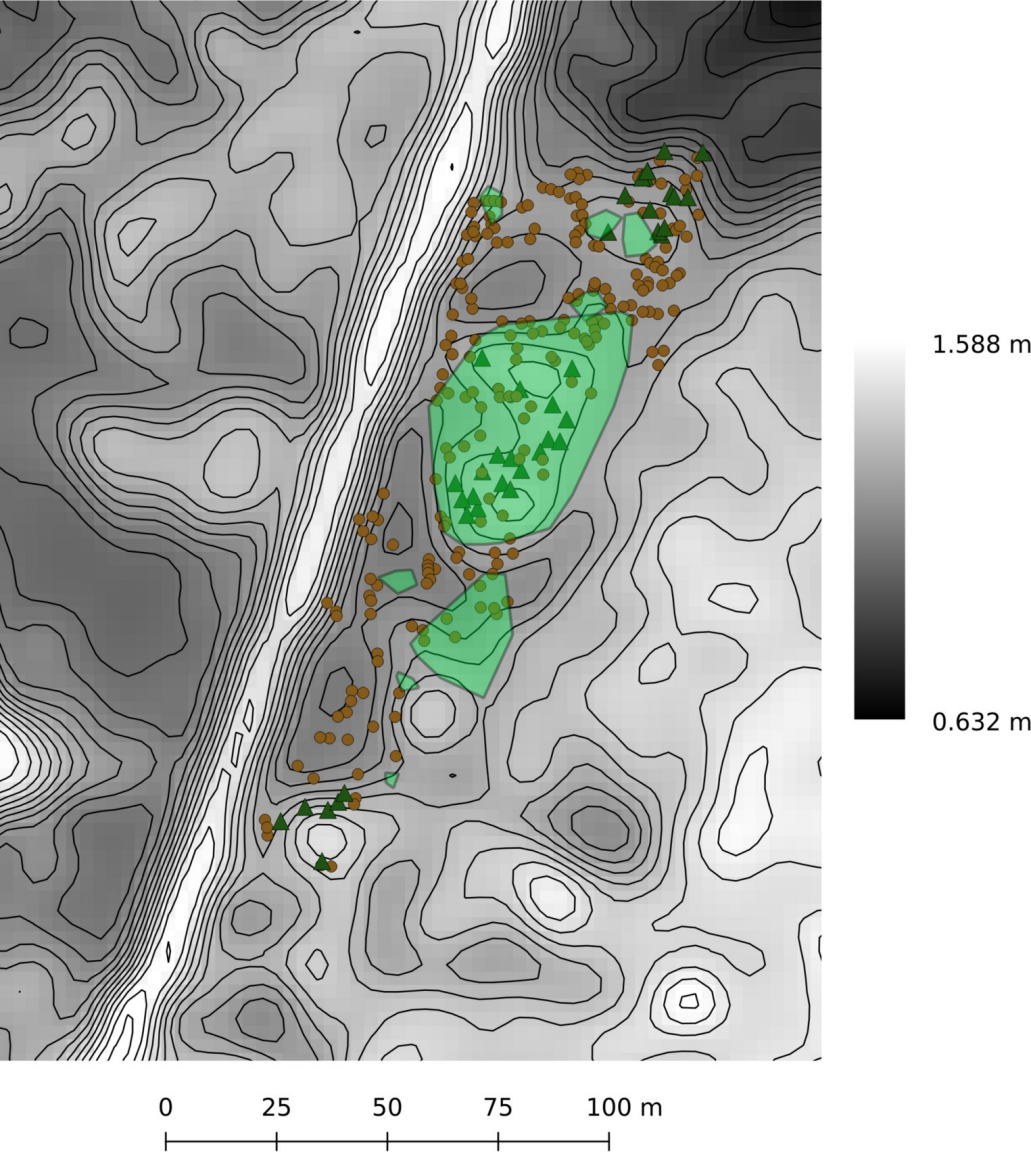
Topographic surveys. Points collected in the hollow topographic survey. Green triangles represent live trees while brown circles represent dead trees. The transparent green regions are the convex hulls of points collected within the sapling clusters. The shaded elevation data comes from a smoothed version of the LiDAR DEM. Contours are 5 cm apart.

The GPS points collected in the hollow topographic survey are presented in Fig 2. In the hollow site, dead trees can be found at all elevations, suggesting that mature forest once covered the hollow site. Live, mature trees are found only on the hummocks. Saplings are found clustered on the hummocks with no saplings in the hollows. In the hillslope site, the live, mature trees are found starting just past the end of the shrub zone. The boundary between the marsh and forest is located at 1.16 ± 0.14 m. The sharp sapling boundary is found around 35 m into the pine forest from those first mature trees. The sapling boundary at the hillslope site is at 1.24 ± 0.13 m NAVD88 while the sapling boundary at the hollow site is at 1.11 ± 0.14 m NAVD88 (mean ± two standard deviations for all reported elevations).

### Groundwater wells

**Fig 3:**
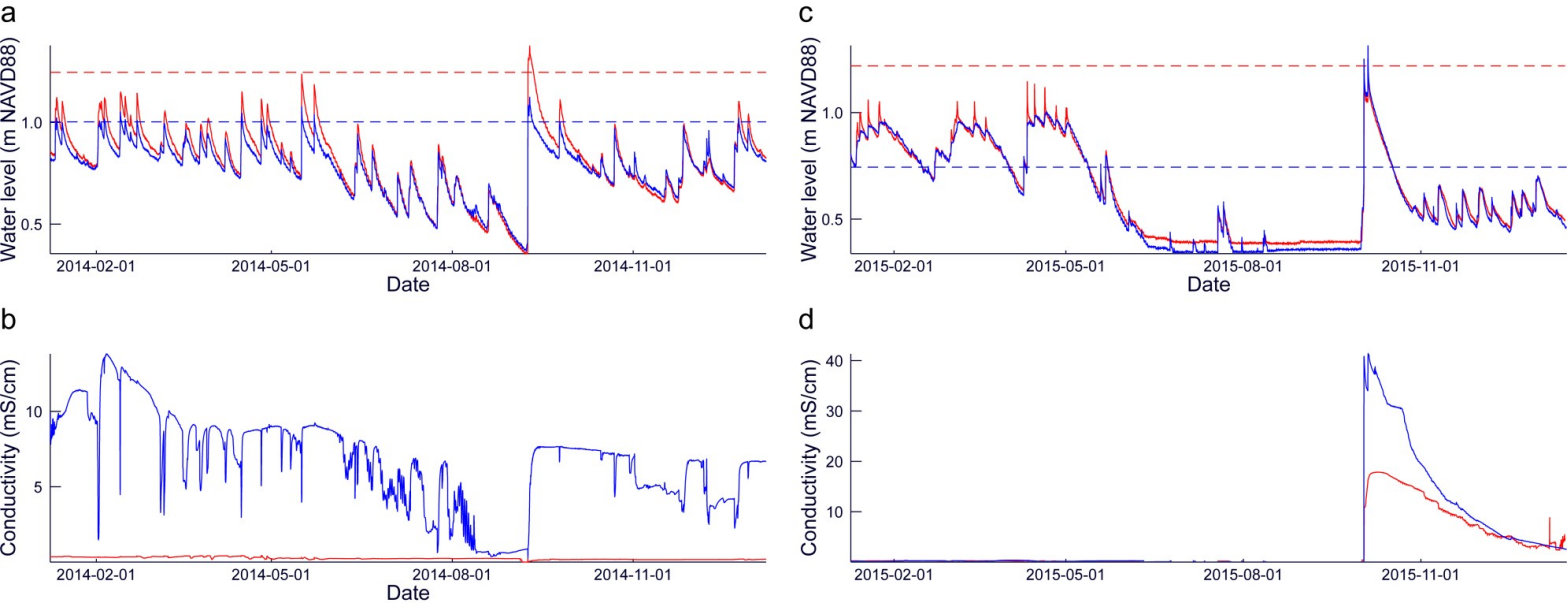
Groundwater wells. (**A)** Water level in the hillslope wells (red: upper; blue: lower). The dashed lines represent the ground surface at the well with the corresponding color. (**B**) Conductivity in the hillslope wells. The colors match the water level plot. (**C**) Water level in the hollow wells (red: hummock; blue: hollow). The dashed lines represent the ground surface at the well with the corresponding color. (**D**) Conductivity in the hollow wells. The colors match the water level plot.

The data from the CTD sensors in the groundwater wells is shown in Fig 3. At both sites, the fluctuations in water level are driven almost entirely by precipitation with a characteristic jump and recession following storm events. Smaller, daily cycles in water level driven by evapotranspiration can also be observed (S3 Fig). Semidiurnal fluctuations, which would be driven by tides, are notably absent in all of the groundwater well data.

#### Hillslope site

The water table in the hillslope wells is normally below the ground surface, flooding only during large storm events, with the lower well flooding more frequently. The water table in the lower well is usually below that of the upper well, indicating the presence of a groundwater mound that follows the surface topography and downslope flow of groundwater. As the water table is drawn down following storms, the water table flattens out and the water level in the two wells converges.

The upper well electrical conductivity is at the lower detection limit for the sensor, and the groundwater underlying the pine saplings is fresh throughout the year. In the lower well, conductivity decreases temporarily during storms, suggesting that brief inputs of freshwater from precipitation make it into the well before mixing with the saline groundwater. As the water table is drawn down in summer, the conductivity decreases, suggesting the migration of the freshwater-saltwater interface down slope.

A large storm on September 8, 2014 at the hillslope site brought increased salinity to the lower well. The storm surge associated with this storm could have been large enough to inundate the lower well directly (maximum water level at the NOAA Kiptopeke station 0.847 m NAVD88 and 1.176 m NAVD88 at the NOAA Wachapreague Station), and the water level in both wells rose above the ground surface. The upper well conductivity does not increase during this storm, so the inputs to the upper well come from precipitation rather than storm surge. The salinity in the lower well remains high after this storm indicating the movement of the freshwater-saltwater interface upslope. Later storm events tend to freshen the lower well, as at the beginning of the record.

#### Hollow site

The water table at the hollow is above the ground surface throughout the winter and spring before being drawn down in the summer. The water table at the hummock is below ground throughout the observation period. The water level in the two wells responds differently to storms, with the hummock well having a sharp convex recession and the hollow well having a slower concave recession. These different responses cause the water level in the hummock well to fall below the water level in the hollow well. The water level is drawn down during the summer below the elevation of both of the sensors.

A storm complex associated with Hurricane Joaquin in the Atlantic struck the Eastern Shore of Virginia on October 2, 2015, and was captured in the hummock-and-hollow site well data. The electrical conductivity in both hummock and hollow wells was negligible throughout the year until the October storm. The conductivity in the hollow well increased during the storm to twice that of the hummock well. The conductivity begins decreasing after the storm, but the hollow well remains more saline than the hummock well until approximately one month after the storm. Other storms throughout the year do not increase the salinity.

### Sea-level rise and persistent zone analysis

**Fig 4:**
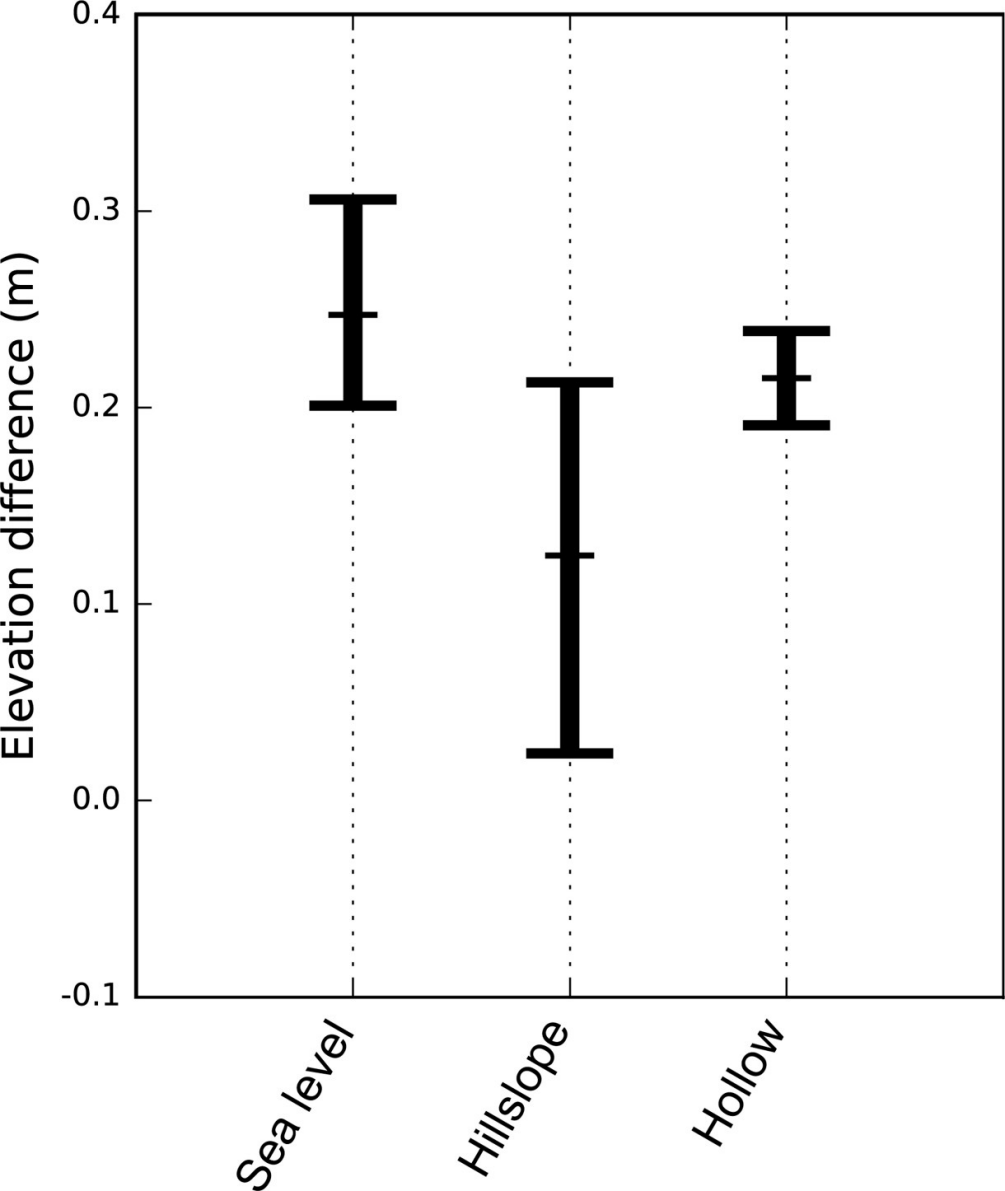
Sea-level rise and persistence zone analysis. Point estimates and confidence intervals for the sea level difference between the year of forest establishment and Hurricane Isabel (2003) and the elevation differences between the lowest trees and the saplings at the hillslope and hollow site.

The trees on the lower boundary of the hillslope site have a median year of establishment of 1939 and a range from 1931 to 1956. This produces a sea-level rise estimate of 0.247 m between forest establishment and the window around Hurricane Isabel (2000–2006) with a 95% confidence interval of 0.201–0.306 m. The elevation difference between the lowest mature trees and the saplings at the hillslope site is 0.124 m with a 95% confidence interval of 0.024–0.213 m and that between the lowest dead trees and the saplings at the hollow site is 0.215 m with a 95% confidence interval of 0.191–0.239 m (Fig 4). There is no significant difference between the increase in sea level since forest establishment and the elevation difference between the lower forest boundary and the saplings at the hillslope site or between the lowest dead trees and the saplings at the hollow site. There is, however, a large amount of variability in the elevation differences at the hillslope site, which is due largely to topographic variability of the mature forest boundary.

**Fig 5:**
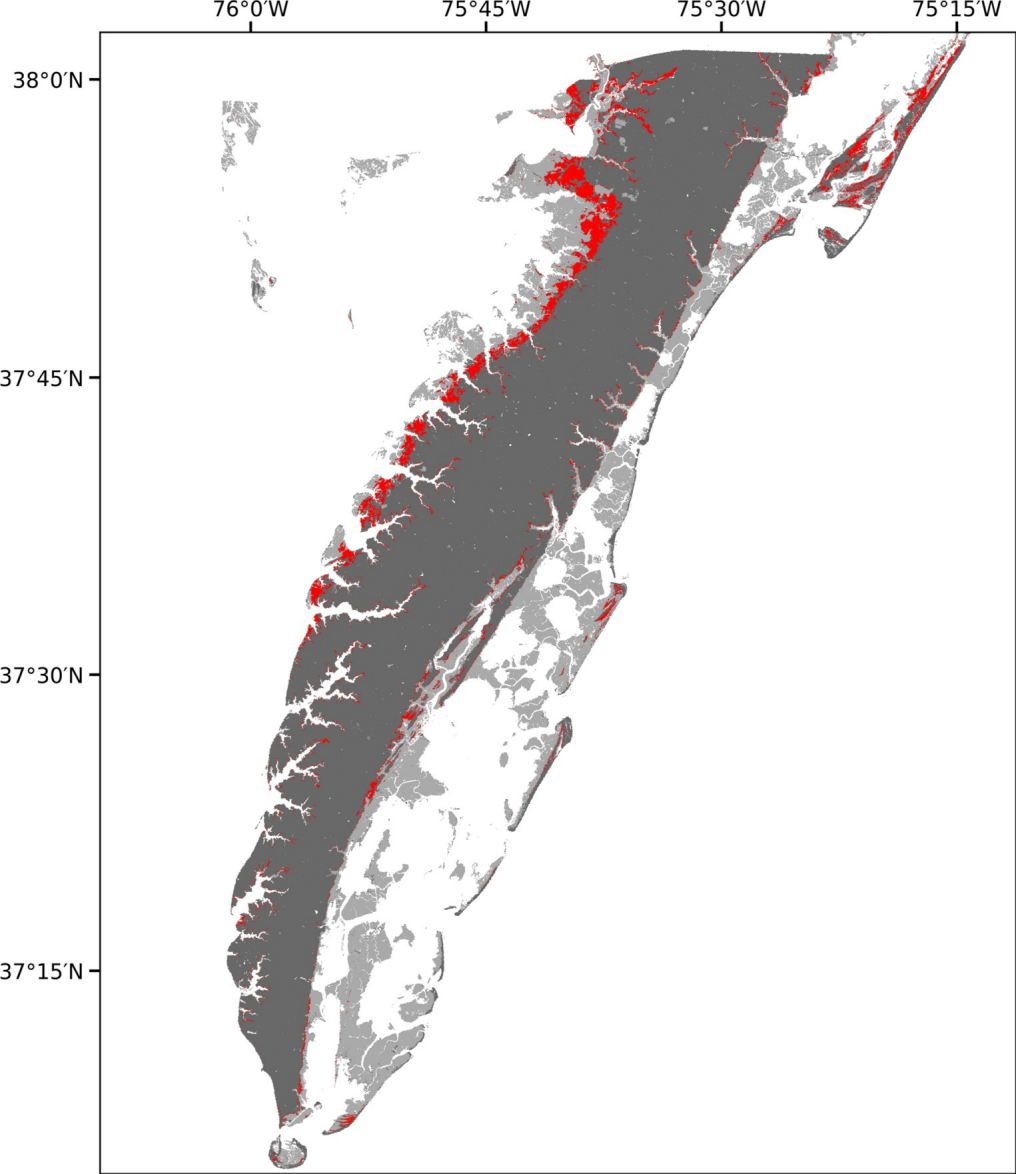
Remote sensing. Map of potential forest dieback based on the elevation of the mean elevation sapling boundary at the hillslope site. Light gray—marsh and shrub/scrub; Dark gray—upland; red—forest within the persistent zone.

Remotely-sensed topographic and land cover data suggests that between 11% and 20% of the forested land area lies within the persistent zone (Fig 5). Most of this area is on the western, Chesapeake Bay, coast of the Eastern Shore because the lower topographic slopes of that side causes an equivalent elevation difference to represent a large horizontal area.

## Discussion

The hydrology of the two sites is markedly different, despite being only 100 m apart. The hillslope site is inundated only by very large storms, and the lower part of the forest regularly stands over saline groundwater. The hollow site is inundated seasonally, and saline water is introduced only by very large storm surges. The hydrological processes that lead to demographic patterning in the pine forests at each of the sites are therefore very different. The hillslope site zonation is controlled by the dynamics of the subsurface freshwater-saltwater interface and by salinity introduced by storm surges. The hollow site zonation is controlled primarily by the seasonal fluctuations in water depth in the hollow with a possible secondary role for salinity during storm surges. The absolute elevations of the two regeneration boundaries are determined by the local hydrological setting. The lower elevation of the regeneration boundary at the hollow site indicates that it is more tolerant to flooding, perhaps because the water at the hollow site is consistently fresher than the water at the hillslope site. However, the similarity in the persistent zone size at both sites suggests that, while local processes control the absolute position of the marsh-forest boundary, a larger-scale driver, namely sea-level rise, controls the vegetation zonation observed in the boundary.

**Fig 6:**
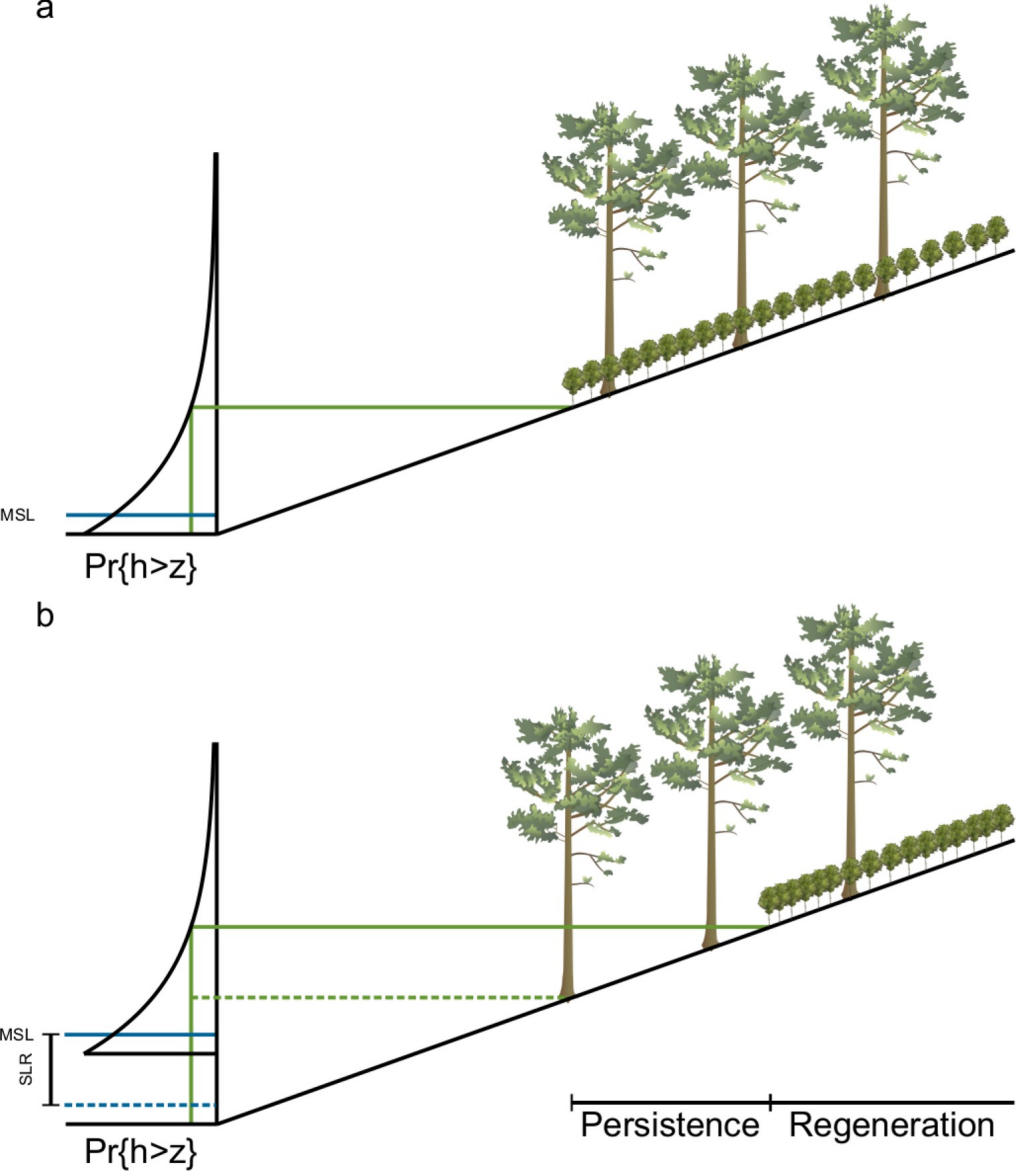
Proposed conceptual model. (**A**) Landscape at time of forest establishment. The distribution on the left is a schematic illustration of a water level exceedance probability function. Mean sea level (MSL) is labeled (solid blue line). A threshold exceedance probability for regeneration is mapped onto a threshold elevation and horizontal position (solid green lines). Below this elevation, young trees cannot grow. (**B**) Landscape after sea level rise. The threshold probability remains constant, but the distribution of water levels changes with sea level (SLR; initial MSL labeled with dashed blue line). so that the regeneration boundary must move upslope (from the dashed green line to the solid green line). Figure reproduced from [34].

These observations and data are consistent with a conceptual model for coastal forest retreat at the hillslope site illustrated in Fig 6 [34]. The hydrology of the site is characterized by an exceedance probability for water level. Pr{h>z} is the probability that the water level, h, exceeds the elevation, z, in a given year. The exact form of this distribution is unique to each site. Because it is defined as one minus the cumulative distribution function of water levels (Pr{h≤z}), it is necessarily monotonically decreasing in z, and there exists a unique elevation for a given probability of observing a water level at or above that elevation. The inverse of the exceedance probability is the return time for a flood that inundates the hillslope up to z. The tolerance of young trees to flooding is modeled as a threshold exceedance probability above which they cannot develop. This threshold is a physiological limit to seedling growth modulated by a term representing local ecohydrological processes. Two stands with the same physiological limit and water level distribution may have different thresholds if fresh groundwater inputs enable seedlings to grow at a lower elevation at one of the sites.

Given the local water level distribution and the topography of the site, this threshold probability maps onto a regeneration boundary, which marks the lowest elevation at which young trees can grow. As sea level rises, the water level distribution moves upward. If neither the shape of the distribution nor the threshold exceedance probability for young trees changes, the regeneration boundary moves upslope at the same rate as sea level rises (Fig 6B). Trees that established at the initial regeneration boundary have matured and, because they are less sensitive to environmental stress, may persist below the new regeneration boundary, forming the persistent zone. The boundary between the forest and the shrubs and marsh below it moves when these mature trees die. Eventually sea level rise will cause regular flooding with saline water, which will lead to mortality. However, under this ratchet model, additional sea level rise is not necessary to result in forest loss. Extreme events like storms such as Hurricane Isabel, which killed many of the mature trees at the hollow site, can kill trees in the persistent zone and lead to forest retreat. The difference in elevation between the lowest mature trees and the present regeneration boundary is equal to the amount of sea-level rise since the establishment of those trees.

This conceptual model explains the similarity in the persistent zone width despite the hydrological variability between the two sites. All the ecohydrological processes that structure the vegetation at a particular site are captured in a single threshold probability. As long as that probability remains constant at the site, this model predicts that the zone of persistence will span an elevation corresponding to the amount of sea-level rise since the establishment of the lowest mature trees, regardless of the actual value of the tolerance. The absolute value of the tolerance determines the elevation of the forest zones relative to sea-level rise, which explains why the elevations of the sapling boundaries at the hillslope and hollow sites differ by nearly 20 cm. Local hydrological processes expose the saplings at the hollow site to less stress than they experience at the hillslope site, and they grow at a lower elevation.

An increase in water level variability, which accompanies an increase in storminess, for instance, can also drive migration of the regeneration boundary (S4 Fig). As the variance of the water level distribution increases, higher water levels become more likely, and the elevation at which the threshold exceedance probability is reached increases. If the variance of water levels increases after the low trees establish, then the regeneration boundary will move upwards, even in the absence of sea-level rise.

A key assumption of this model is that the tolerance threshold of trees to flooding is constant in time. However, this tolerance could depend on the cumulative amount of stress experienced by the trees from flooding as well as from droughts, extreme temperatures, or disease. It could also depend on competitive or facilitative ecological interactions between trees or between trees and the grass and shrub species of the understory [23]. We therefore expect fluctuations around the persistent zone size that would be estimated given a constant threshold exceedance probability. Sites which have become more stressed since the trees established should have a larger persistent zone size than estimated from the sea-level rise, while those which have become less stressed should have a smaller zone size. These fluctuations could be studied by comparing the deviation from the predicted persistent zone size to tree ring data or, where they exist, to time series of forest health data.

The conceptual model proposed here is a prototypical ecological ratchet in the sense of Jackson et al. [28]. The migration of the regeneration boundary is ultimately driven by the slow press of sea-level rise, but the actual movement of the marsh-forest boundary happens in pulses when events kill mature trees in the persistent zone. On long time scales, i.e. centuries, the migration of the marsh-forest boundary integrates over these pulses and is likely to resemble a deterministic migration upslope as in the model of Kirwan et al [12].

The observed zonation is consistent with a model that expresses the migration of the regeneration boundary in terms of a threshold exceedance probability that captures the tolerance of trees to flooding. A first-order approximation of the size of this persistent zone, which is obtained under the assumption of a constant threshold exceedance probability, is the amount of sea-level rise experienced since the forest establishment. This proxy gives an estimate for persistent zone size that is insensitive to the particular setting of any given coastal forest and that should apply in a wide variety of settings. Further research is needed to determine the extent of persistent zones in other coastal forests and to identify the sources of variability in persistent zone size. Another test of the hypothesized relationship between sea-level rise and forest persistence would be the reproduction of both the qualitative demographic zonation and the quantitative zone sizes in mechanistic models of coastal forests forced by storms and sea-level rise. The key physiological process to include in these models is the age-dependent response of trees to the stresses induced by sea-level rise without which there is no difference between the persistence and regeneration niche, which is required to produce an ecological ratchet.

We have shown that there exists a persistent zone of coastal pine forest at the Eastern Shore of Virginia National Wildlife Refuge site. Young trees cannot establish in this zone because they are particularly sensitive to flooding or salinity stresses, but less-sensitive older trees, which established approximately 80 years ago, can survive. This persistent zone is vulnerable to extreme events as this portion of the forest is unable to regenerate when mature trees are killed by events such as storms. Up to one-fifth of the total forested land area on the Eastern Shore could lie within this persistent zone and is thus vulnerable to sudden dieback after a severe storm.

## Supporting information

S1 Fig Vegetation surveys. (**A**) Quadrats as classified in the field. White: low pine density; Blue: high pine density. (**B**) Quadrats classified by visual interpretation of aerial imagery. (**C**) Quadrats as classified by the logistic regression model. High-resolution orthoimagery courtesy of the United States Geological Survey (image date: February 14, 2013).
(EPS)

S2 Fig NDVI time series. (**A**) the hollow site and (**B**) the hillslope site. The red vertical line marks the date of landfall of Hurricane Isabel (September 18, 2003).
(EPS)

S3 Fig Subset of the groundwater level data. (**A**) Water level in the hillslope wells (red: upper; blue: lower). (**B**) Conductivity in the hillslope wells. The colors match the water level plot. (**C**) Water level in the hollow wells (red: hummock; blue: hollow). (**D**) Conductivity in the hollow wells.
(EPS)

S4 Fig Conceptual model under an increase in variance. The same as Fig 6 in the main text except with the addition of (**C**) Landscape after an increase in the variance of the water level distribution. The mean sea level increases slightly, but the increase in variance drives a retreat of the regeneration boundary as large as that seen in (**B**) with only sea-level rise. Figure reproduced from [34].
(EPS)

S1 Table Logistic model selection results. (DOCX)

S2 Table Parameters for the selected logistic regression model. (DOCX)

S3 Table Groundwater well parameters. (DOCX)

S1 Text Supplementary methods. (DOCX)
