# Supplementary material for: Sea-level rise and storm surges structure coastal forests into persistence and regeneration niches
Source: PLoS One. 2019 May 2;14(5):e0215977. doi: 10.1371/journal.pone.0215977 (PMC6497265; doi:10.1371/journal.pone.0215977)
Supplement: S1 Table — (DOCX) [file pone.0215977.s005.docx]

| Model | AIC |
| --- | --- |
| Intercept only | 34.671 |
| Mean elevation | 15.519 |
| Mean elevation and standard deviation | 17.125 |
