# Supplementary material for: Sea-level rise and storm surges structure coastal forests into persistence and regeneration niches
Source: PLoS One. 2019 May 2;14(5):e0215977. doi: 10.1371/journal.pone.0215977 (PMC6497265; doi:10.1371/journal.pone.0215977)
Supplement: S2 Table — (DOCX) [file pone.0215977.s006.docx]

| Parameter | Estimate | P-value |
| --- | --- | --- |
| Intercept | -29.9483 | 0.0110 |
| Mean elevation | 30.5179 | 0.0094 |
