# Supplementary material for: Sea-level rise and storm surges structure coastal forests into persistence and regeneration niches
Source: PLoS One. 2019 May 2;14(5):e0215977. doi: 10.1371/journal.pone.0215977 (PMC6497265; doi:10.1371/journal.pone.0215977)
Supplement: S3 Table — (DOCX) [file pone.0215977.s007.docx]

| Well | Depth from surface to sensor | Elevation of sensor (m NAVD88) | Deployment dates |
| --- | --- | --- | --- |
| Hillslope – lower | 0.80 | -0.5072 | 2014-01-07 – 2015-01-09 |
| Hillslope – upper | 0.91 | -0.3344 | 2014-01-06 – 2015-01-09 |
| Hollow – lower | 0.37 | 0.374 | 2015-01-09 – 2016-01-16 |
| Hollow – upper | 0.81 | 0.409 | 2015-01-09 – 2016-01-16 |
