# Supplementary material for: Sea-level rise and storm surges structure coastal forests into persistence and regeneration niches
Source: PLoS One. 2019 May 2;14(5):e0215977. doi: 10.1371/journal.pone.0215977 (PMC6497265; doi:10.1371/journal.pone.0215977)
Supplement: S1 Text — (DOCX) [file pone.0215977.s008.docx]

# Supplementary Methods

## Vegetation survey

A survey of vegetation types was conducted in the hollow site (S1 Fig). A grid of plots 10 m on a side was laid out with five transects running parallel to Wise Point Road and ten quadrats on each transect. A random selection of 25 of the 50 quadrats was created by sampling five boxes from each transect. The range of a variogram estimated from the topographic survey procedure is 42.3 m, suggesting that our plot size is below the scale of variability of the elevation and that each quadrat samples a roughly flat region. Within each sampled box, the percent cover of grassy species was recorded. Individuals of woody plant species were counted. Furthermore, the location of pine trees and saplings was recorded within each box to provide some information on spatial patterns below the scale of the boxes. Each quadrat of the vegetation survey was classified in the field as having a high pine density or a low pine density with the threshold between the two classes being roughly twenty trees within the quadrat. Both saplings and mature trees were included in the density estimate.

A logistic regression was used to predict the density of pine trees within each quadrat based on elevation and topographic roughness. Within each quadrat, the average elevation and standard deviation of elevation were calculated from the LiDAR-based digital elevation model. The elevation provides an indication of the flooding frequency of the quadrat while the standard deviation provides an estimate of the topographic roughness and tendency for ponding to occur within the quadrat, which helps characterize ecohydrological processes at a scale below that of the quadrat.

The logistic model was validated using remotely sensed data. The quadrat grid was located on four-band aerial orthoimagery of the site taken in 2009. Each of the 50 quadrats in the grid was classified into high or low tree density by visual inspection of the orthoimage, independently of the field classification.

Model selection using the Akaike information criterion suggests that logistic regression model which includes only the mean elevation in each quadrat is more parsimonious than the model which uses both the mean and the standard deviation of elevation (S1 Table). This is consistent with the finding that the quadrat length scale is below the dominant scale of topographic variability. Using the selected model (estimated parameters given in S2 Table) we calculate a threshold as the elevation where predicted probability of the high-density class is 0.5. This threshold is found to be 0.98. The validation quadrats were then classified based on this elevation threshold (S1 Fig) and compared to the classification based on aerial photographs.

Out of the 50 quadrats, the logistic model correctly predicts the presence or absence of pine trees in 46 quadrats. Two of the misidentified quadrats were included in the field vegetation survey, and one of those two was incorrectly classified in the aerial photographs. This quadrat does contain pine trees, according to the field survey, but they are not detectable in the aerial image. In this case, the model agrees with the field survey and not the aerial imagery. On the two mislabeled quadrats at the southern end of the site (one of which was part of the field survey), the model predicted there would not be trees when pine saplings are present. However, the saplings in these quadrats were observed in the field to have browning needles during the growing season and are under apparent stress, which would suggest that this represents a relaxation back to the depression vegetation state after trees were able to establish on this part of the site. This leaves one quadrat which the model predicts to have pine trees which does not appear to have trees in the aerial image and which was not sampled during the field survey. However, the topographic survey does record several GPS points associated with pine saplings in one corner of the box. We are therefore confident that the logistic model and the elevation threshold of 0.98 m NAVD88 perform fairly well at identifying the locations of pine trees within the site. This elevation threshold was used in the main text to separate the dead trees at low elevations from dead trees at higher elevations. The elevations of the dead trees below this threshold and of the saplings, which exist almost entirely above this threshold, were used to compute the persistence zone size at the hollow site.

## Remote sensing

A time series of Landsat satellite imagery from 1982 to 2016 was used to observe the recent history of the forest. 694 Landsat scenes (path 14, row 34) collected by the Landsat 4 and 5 TM sensor and the Landsat 7 ETM+ sensor with less than 20% cloud cover were identified, and the corresponding surface reflectance products were downloaded. Clouds remaining in the images were masked out with the Fmask algorithm (31), and only clear pixels were included in the analysis. From these surface reflectances, the Normalized Difference Vegetation Index (NDVI) was calculated as

NDVI = (*NIR – Red)/(NIR + Red)*

where (*Red)* is the reflectance in the red (TM/ETM+ band 3) and (*NIR)* is the reflectance in the near-infrared (TM/ETM+ band 4). NDVI is commonly used to assess the health of vegetated landscapes.

The time series for two 30m x 30m Landsat pixels is shown in S2 Fig. Following Hurricane Isabel in September 2003, the NDVI declines at both the hollow and hillslope sites. Furthermore, Isabel is the only such event observed in the 34 year time series. The decline at the hollow site is more pronounced, and the forest does not appear to recover as well at the hollow site as it does at the hillslope site. We take this sharp decline as evidence that most of the mortality observed in the hollow site was caused by Hurricane Isabel. We use this observation to justify choosing Hurricane Isabel as the date at which the saplings in the hillslope and hollow sites established.

## Groundwater wells

Two groundwater wells were installed at the hillslope (January 2014—January 2015) and at the hollow site (January 2015—January 2016). Each well was augured to a depth of up to a meter and No. 10 slotted well screen was inserted into the well. A conductivity-temperature-depth sensor (CTD-Diver; SWS) was hung from a line in each well, and a third pressure sensor (Baro-Diver) was affixed to a nearby tree at approximately two meters height. The wells were surveyed with an RTK-GPS (Topcon), and the elevation of the sensor was calculated from the length of line in the well. The relevant parameters are given in S3 Table.

For reference, the mean sea level at the nearby NOAA tide station at Kiptopeke (8632200) is -0.146 m (NAVD88).

A subset of the water level from each site is given in S3 Fig to illustrate fine-scale fluctuations in water level and conductivity. Diurnal water level fluctuations in phase with the day suggest that these are driven by evapotranspiration rather than tides.

## The effect of storminess on persistent zones

An increase in storm frequency or intensity that increases the variance of the water level distribution results in a situation illustrated in S4 Fig. A small increase in the mean sea level accompanied by increased variability drives the regeneration boundary upslope as far as a larger increase in sea level without increased variability.
